# Integration of patient and clinician narratives into systematic reviews: An applied proof of concept

**DOI:** 10.1017/cts.2021.2

**Published:** 2021-01-19

**Authors:** Vivian Christensen, Khaya Clark, Stephanie Tallett, Emily A. Kenyon, Steven K. Dobscha

**Affiliations:** 1 VA Evidence Synthesis Program Coordinating Center, VA Portland Health Care System, Portland, OR, USA; 2 Oregon Clinical and Translational Research Institute (OCTRI), Oregon Health and Science University, Portland, OR, USA; 3 Center to Improve Veteran Involvement in Care (CIVIC), VA Portland Health Care System, Portland, OR, USA; 4 Department of Veterans Affairs, Contractor zCore Business Solutions, Inc., National Center for Rehabilitative Auditory Research, Portland, OR, USA; 5 Department of Defense, Hearing Center of Excellence, San Antonio, TX, USA; 6 Office of Human Factors, VA Portland Health Care System, Portland, OR, USA; 7 Department of Psychiatry, Oregon Health and Science University, Portland, OR, USA

**Keywords:** Deprescribing, systematic review, qualitative methodology

## Abstract

Health systems currently underutilize systematic reviews. Here, we describe a proof of concept project designed to augment the standard systematic review process by presenting qualitative information as a companion to a review on deprescribing interventions. We conducted a thematic analysis of semi-structured interviews with Veterans Health Administration clinicians and Veterans to describe first-hand experiences of engaging in the deprescribing process. Qualitative findings were incorporated into an interactive, web-based product designed to supplement the systematic review report. Preliminary evaluation suggests that integration of narratives as a companion to systematic reviews is of interest to frontline clinicians, researchers, and health system administrators.

## Introduction

Studies have found that health care managers and policy-makers underutilize traditional systematic reviews, in part, due to lack of specific clinical recommendations and implementation strategies [1–4]. Traditional systematic reviews, which prioritize findings from randomized controlled trials, are often insufficient in addressing contextual details that can make implementation more likely to succeed [5,6]. Further, while investigators have demonstrated that including patient perspectives in quality improvement efforts can increase clinically actionable findings [7], the “voices” of patients and frontline clinicians are typically absent from evidence-synthesis processes and reports. The inclusion of qualitative data as a method of supplementing literature findings, providing context needed for implementation of findings and conveying the voices of patients and clinicians, has potential to increase utility, and ultimately, impact of systematic reviews. Narratives also have the potential to enrich systematic reviews by providing experiential information often not captured in traditional reviews and engage audiences who may not be as invested in primarily quantitative information.

To understand the potential impact of augmenting the standard systematic review process by integrating qualitative information, we developed a proof of concept project on the topic of deprescribing. Our project had three goals: to develop narratives, using in-depth interviews, highlighting the perspectives and experiences of Veterans and their clinicians who have experienced the process of deprescribing first-hand; to develop an interactive multimedia product as a method of presenting our findings as a supplement to a traditional systematic review being conducted by the VHA Evidence Synthesis Program (ESP); and to conduct a preliminary evaluation of the value, impact, and feasibility of such a product.

Deprescribing interventions are designed to address polypharmacy, defined as the concurrent use of multiple medications by a patient. Over 40% of adults aged 65 and older use five or more prescription medications [8]. Polypharmacy is associated with an increased risk of adverse drug events, drug–drug interactions, medication nonadherence [9] as well as increased falls, dementia, and mortality [10]. As such, mitigating polypharmacy among older adults is a high priority for clinicians and health systems. Targets for deprescribing interventions include eliminating or reducing medications without clear indication, duplicative medications, and medications that adversely affect cognition and function [10]. Here, we describe the development of and evaluation of the product.

## Methods

This project was designated a quality improvement project by the VHA Medical Center where the project was conducted. We employed a snowball sampling method to identify clinicians representing primary care, geriatrics, mental health, and clinical pharmacy who had engaged with patients in the deprescribing process. We initially reached out to several colleagues working in these areas and asked them to identify others who might be willing to participate in interviews. Verbal consent was provided by each participant prior to initiation of the interview. During each interview, physicians were encouraged to identify a patient with whom they had initiated deprescribing, as well as identify other staff who had also engaged with that patient in deprescribing efforts. Our goal was to develop narratives or stories which serve as case studies and portray patient–clinician interactions around deprescribing from multiple perspectives. Each clinician we interviewed was provided with a letter describing our project including our contact information to give to his or her prospective patient, stressing that participation was voluntary. At the end of each interview, clinicians were asked to identify colleagues whom they felt would be willing to participate.

We developed semi-structured interview guides for clinicians and patients (Table 1; Supplement 1: Clinician Interview Guide; Supplement 2: Veteran Interview Guide) based on information from three sources: 1) a review of existing literature regarding facilitators and barriers to deprescribing; 2) a focus group discussion with the Veteran Engagement Group of the VHA Health Services Research & Development (HSR&D) Center to Improve Veteran Involvement in Care, a standing group of Veterans who provide guidance to researchers to promote Veteran-centered research design; and 3) discussions with two VA internal medicine physicians known by the project team who had deprescribing experience and agreed to initial scoping interviews in which potential interview questions were discussed.

**Table 1.** Key domains of semi-structured interview guides

Clinician interview guide
• Provider characteristics: Current VHA role, length of time practicing at VHA, current panel size
• Description of deprescribing process with a specific patient (narrative)
• Provider/patient goal(s) of deprescribing, process, patient involvement in decision-making, what went well (facilitators), challenges encountered (barriers), end result
• Reflections on the deprescribing process (what would have made the process go more smoothly)
• Beliefs, attitudes, and knowledge about deprescribing
• Advice to other clinicians (key strategies that facilitate successful deprescribing)
Veteran interview guide
• Veteran characteristics: length of time as a patient at the VHA, general health questions, number of medications currently taking
• Experiences working with their doctor and other care providers (e.g., clinical pharmacist) to reduce, stop, or lower the dose of a medication (narrative)
• Patient’s goal(s) of deprescribing, attitudes and perceptions about deprescribing, involvement in decision-making, what went well (facilitators), challenges encountered (barriers), social/family support during the process, end result
• Reflections on the deprescribing process (what could have been done to make the process go more smoothly)

All interviews were conducted in-person between March and August 2019, lasted 30–60 minutes, and were audio-recorded and transcribed verbatim. Using an inductive narrative approach, we conducted a thematic analysis [11] of semi-structured interviews with VHA clinicians and Veterans to describe first-hand experiences of engaging in the deprescribing process. This approach seeks to identify the context and meaning of experiences as told by the storyteller through narrative. The first four transcripts (two provider and two patient interviews) were dual coded by two PhD sociologists with expertise in qualitative methodology, implementation science, and systematic review research, focusing on the core elements of each narrative. The remaining transcripts were independently coded. Using an iterative process, identified themes were deliberated until consensus was reached. Atlas.ti was used to code and analyze interview data.

## Results

We interviewed 10 healthcare providers, including clinicians representing primary care (*n* = 4), geriatrics (*n* = 2), and mental health (*n* = 2). In addition, we interviewed a clinical pharmacist and a licensed nurse practitioner who had engaged with identified patients around deprescribing. All Veterans who expressed interest were interviewed, including three participants who approached us about wanting to share their deprescribing stories. In total, we interviewed five Veterans, all of whom were white, male, and over the age of 65 years. All clinicians who were contacted, and all but one Veteran who were asked to participate, completed an interview.

We were able to develop two complete case studies (i.e., patient and clinician or others on patient care team), which are presented as two stories in the product. These stories are based on excerpts from the interview data in which both the Veteran and their clinician(s) were interviewed about the same deprescribing effort. Both facilitators and barriers to deprescribing are portrayed using actual participant quotes. To protect the identity of those who participated, we used pseudonyms and publicly available stock photos to illustrate experiences; all but one of the photographs used reflect the gender, rough age, and race of the actual participants. An “Advice from Veterans and Clinicians” page was added based on themes that emerged from our analysis across all 15 interviews. For this feature, a drop-down menu appears that includes exemplar quotes for each theme from both clinicians and Veterans, see https://www.hsrd.research.va.gov/publications/esp/deprescribing/ to view the product.

Thematic analysis of the interviews revealed several factors which lead to successful deprescribing, including establishing trust, communicating the rationale for deprescribing, understanding patients’ health goals and preferences, practicing shared decision-making, providing options for symptom management, and committing to working with patients over time. Identified barriers included challenges with coordinating care, lack of patient understanding of risks associated with polypharmacy, and in some cases, limited alternative treatment options.

### Preliminary Project Evaluation

We experienced several challenges as we developed this product: The first was the limited number of complete case studies we were able to capture. Physicians were often reluctant to reach out to a particular patient on our behalf, instead preferring to summarize deprescribing interactions with multiple patients. Similarly, patients often discussed past deprescribing experiences across interactions with multiple providers. We encountered additional challenges in creating the product, including the need to bring in high-level expertise to design and build a high-quality interactive product, and the need to conform to VHA’s web page technical requirements.

In order to examine the potential value and impact of the product, we initially solicited feedback from subject matter experts who served as external peer reviewers for the VHA ESP systematic review entitled “Deprescribing for Older Veterans.” These subject matter experts (*n* = 7), including four who currently have clinical responsibilities within VHA, were asked for feedback about the content of the product, relevancy of the product to their professional activities, and whether the product added to their knowledge base on the topic. Overwhelmingly, responses were very positive, and using a five-point Likert scale, all strongly agreed or agreed to a series of statements describing its utility. Moreover, all reviewers believed that the product added value to the traditional systematic review. Additional open-ended feedback was provided by each reviewer. One reviewer commented: “The very real clinical scenarios, which describe why deprescribing is important, capture the difficulties of deprescribing and, through the stories, provide useful guidance regarding approaching these issues. The link to summarizing key points of advice then makes the message actionable.” Another reviewer wrote: “It illustrates the real-world challenges associated with deprescribing. This is not an easy task and must be done including the Veteran in decisions. These points came across beautifully in the presentation.” A third reviewer stated: “Seeing the actual process of deprescribing from the various stakeholders was extremely compelling. I thought the product was very relevant and enhanced the review.”

To further enhance our preliminary evaluation, we queried 56 audience members who attended a VHA HSR&D Cyberseminar held on April 21, 2020, in which both the ESP systematic review and our narrative project were presented, about the likelihood that they would use such a product in the future. We received anonymous responses from 36 (response rate 64%) audience members (16 clinicians, 16 researchers, and 4 administrators). Using a five-point Likert scale, 62% of clinicians replied that they would be very likely or somewhat likely to use such a product and 38% were unsure. Among researchers, 62% also replied that they would be very likely or somewhat likely to use such a product and 38% were unsure. Among administrators, all four said that they would be very or somewhat likely to use such a product.

## Discussion

To our knowledge, this is the first time that an interactive, web-based product, depicting first-hand experiences of patients and clinicians, has been created to serve as a companion to a traditional systematic review report. The product is designed to portray stories from multiple perspectives that demonstrate barriers and facilitators to deprescribing, and at the same time, help make the report more salient and potentially actionable to audiences of the report. Our preliminary evaluation suggests that integration of narrative into the systematic report presentation is of interest to frontline clinicians, researchers, and health system administrators.

Integration of narrative into the systematic review process can be considered an effort to improve quality of systematic reviews. In a recent article, Grob *et al.* [7] noted that incorporation of narratives of patient experiences in outpatient care into quality improvement efforts shows promise for conveying actionable information to clinicians and administrators. Lin *et al.* [6] recently suggested that integration of health system data with traditional systematic reviews may help overcome decisional uncertainty for healthcare decision-makers by improving applicability of evidence or to support the implementation of the evidence. In our case, the narrative product integrates qualitative rather than quantitative information in an analogous way, that is, in an effort to improve applicability of the review findings and to offer stakeholder perspectives and experiences that may foster implementation.

## Limitations

Our project has several limitations. Our stories were derived from a small number of individuals using a snowball sampling method that could have introduced bias to our findings, and although we identified common themes from the interviews, some important perspectives and experiences may not have been captured. In particular, due to the approach we took to sampling, all five of the patients we interviewed were white, male, and over the age of 65. It would be critical in future efforts to attempt to gather perspectives from a more diverse group of individuals in terms of race/ethnicity, gender, and age. Finally, as noted, this was a proof of concept project. While the findings from our preliminary evaluation are promising, additional work must be done to further evaluate this type of product. In particular, a larger project is needed to compare impacts of the product integrated with a systematic review report versus a systematic review report alone. Potential outcomes might include reach to various audiences and effects on decision-making by administrators, implementation of new care processes, or changes in clinician behaviors, all of which would be challenging to measure. It would also be helpful to evaluate the costs of creating such products to be able to fully appraise their value.

## Supplementary material

For supplementary material accompanying this paper visit https://doi.org/10.1017/cts.2021.2. click here to view supplementary material
