# Supplementary material for: Integration of patient and clinician narratives into systematic reviews: An applied proof of concept
Source: J Clin Transl Sci. 2021 Jan 19;5(1):e78. doi: 10.1017/cts.2021.2 (PMC8111606; doi:10.1017/cts.2021.2)
Supplement: Supplementary file 1 [file S2059866121000029sup.zip › S2059866121000029sup001.docx]

**Deprescribing Clinician Interview Guide**

**Introductions**

Thank you for taking the time to talk with us today. We are conducting a quality improvement project that will generate a companion product for a systematic review being conducted on the effectiveness of deprescribing interventions for older adults. Our aim is to interview clinicians and (ideally) older Veterans (aged >65) who are working with their clinicians on polypharmacy (e.g., lowering the dose of a medication, tapering off a medication). We plan to develop “stories” from the perspectives of the patient, clinician, and perhaps others in the patient’s life (e.g., caregivers, pharmacists). These “stories” will be presented along with the upcoming systematic review on deprescribing. In this project, we are not focused on opioids.

Today, we would like to learn about some of your experiences working with patients on deprescribing; and get your input on what you think might be the best process for recruiting a couple of patients to interview on *their* experiences with deprescribing. Do you have any questions for us about our project?

Before we begin, I would like to talk about the interview process. You can choose to skip any question if you feel uncomfortable. Also, our conversation will be recorded today so that I can accurately capture your comments. When we transcribe the audiotapes, we will remove any identifying information (e.g. names, cities, etc.) so that your comments remain anonymous. Do you have any questions before we begin?

*So to get started with the interview, we need to obtain your verbal consent for the recording. Is it okay with you for us to record this interview?*

[IF YES, START RECORDER]

*I’ve started the recorder. Would you please state your name for the verbal consent process?*

*Do you consent to being recorded to participate in this study?*

*Great, thank you.*

**Background**

1. First, please tell us about your role as a clinician at the VA.

**Probes:**

- How long have you been practicing at the VA?
- In which clinic do you practice?
- How many patients do you follow or how large is your patient panel (primary care)?

1. How do you identify patients who may be candidates for deprescribing?

**Probes:**

- In general, how do your patients react when you bring up the topic?

1. Please tell us about your experience of deprescribing with a specific patient and what that process was like. **Ideally, we would like to hear about a patient who had challenges with the deprescribing process and someone you think might be willing to talk to us.**

**Probes:**

- Please tell us a little bit about this patient (age and health status)?
- What medication did you taper?
- Why did you decide to taper this patient off of the medication?
- What was the ultimate goal of tapering the patient off of the medication?)
- How did you bring up the topic with this particular patient?

1. Tell us a little bit about your patient’s response to your recommendation to stop or to taper the medication(s).

**Probes:**

- How did your patient react to your recommendation to stop the medication?
- Did they agree or disagree?
- What were the patient’s concerns?
- Did the patient participate in the decision or process to taper?
- How did the patient participate in the process?
- Did others participate?
- Was the patient offered alternative treatments or was he/she give options for how the taper was conducted?

1. As you reflect on the deprescribing process with this patient, can you think of anything that would have helped make the process go more smoothly? What would you have done differently?

**Probes:**

- What were the main challenges in the process (e.g., limited time)?
- What went well about the process (e.g., helpful decision aids, clear guidelines, engagement of other team members, motivated patient)?
- Did you follow up with the patient and monitor the progress? If so, how?
- Did you provide additional support or guidance to the patient? If so, what support or guidance did you provide?
- How long did the process take?
- What was the end result of your effort to deprescribe?

**Beliefs, attitudes, and knowledge towards deprescribing (Only ask if there is time)**

1. Where does deprescribing fit into your clinical priorities?
2. How comfortable are you with the process of deprescribing?
3. Please describe any general challenges (not patient specific) you face to deprescribing.
4. Please tell us if there is anything about the deprescribing process that you would like us to know more about.
5. As we mentioned, we would like to identify a potential patient whom you have worked with around deprescribing, or with whom deprescribing is likely indicated but has not yet occurred. **We are very interested in talking to a patient with whom you initiated deprescribing where the end result was not successful.** Please note that the IRB has designated this as a quality improvement project, but we would like your permission to talk to your patients.

**Probes:**

- Would you be willing to talk to them/send a letter on our behalf?

**General question:** If you were to give advice to other clinicians about deprescribing, what would you say about what they can do to make deprescribing manageable or successful?
