# Supplementary material for: Integration of patient and clinician narratives into systematic reviews: An applied proof of concept
Source: J Clin Transl Sci. 2021 Jan 19;5(1):e78. doi: 10.1017/cts.2021.2 (PMC8111606; doi:10.1017/cts.2021.2)
Supplement: Supplementary file 1 [file S2059866121000029sup.zip › S2059866121000029sup002.docx]

**Deprescribing Patient Interview Guide**

**Introductions**

Thank you for taking the time to talk with us today. We are conducting a project focusing on how clinicians, providers, nurses, and pharmacists talk with patients and their families about changing medications. Our aim is to interview Veterans (aged >65) who are working with their clinicians (e.g., to lower the dose of a medication, stop a medication, or reduce the number of medications being taken). We are also interviewing clinicians at the VA about working with their patients on changing medications. We plan to develop “stories” from the perspectives of the patient, clinician, and others in your life (e.g., family members or caregivers) to share with clinicians, researchers, and administrators.

Today, we would like to learn about your experience working with your provider or a pharmacist on changing your medications. Do you have any questions for us about our project?

Before we begin, I would like to talk about the interview process. You can choose to skip any question if you feel uncomfortable. Also, our conversation will be recorded today so that I can accurately capture your comments-- to do this, I will ask for your verbal consent for the recording by asking you to state your first and last name here shortly.

When we transcribe the audiotapes, we will remove any identifying information (e.g. names, cities, etc.) so that your comments remain anonymous. At the end of this interview, I will confirm your mailing address and our team will send you a $30 Amazon gift card.

Before we start, do you have any questions before we begin? Is it ok I start the audio recorder?

[IF YES, START RECORDER]

I’ve started the recorder. Would you please state your name for the verbal consent process?

Do you consent to being recorded to participate in this study?

Great, thank you.

**Demographic questions**

What is your age?

How do you describe your race or ethnicity?

And what about your gender?

What is your highest level of education?

**Background**

1. First, please tell me about how long you have been a patient at the VA.

**Probes:**

- What is the name of your primary care/mental health provider?
- How long have you received care from your current provider?

1. In which branch of the military did you serve?

**Probes:**

- When did you serve?

1. Please tell me what your health is like these days.

**Probes:**

- What kind of medical issues do you have. (Ask only if necessary)
- Do you have any family members or close friends who help you with your health?

1. How many medications do you take daily?

**Probes:**

- Do you have any trouble managing your medications (e.g., cost, frequency of taking your medications)?

1. Thinking back to visit(s) with your doctor (pharmacist), please tell me a little about working with your doctor (pharmacist) to reduce, stop, or lower the dose of a medication.

**Probes:**

- Did you or your provider want to change or stop your medication?
- What medication was stopped?
- How long had you been on this medication?
- What reason did your provider give you for changing your medication, or what motivated you to bring it up with your doctor?
  - What was the reason?

1. Please tell me how you felt about changing or stopping your medication?
2. Did your provider give you any information on the benefits or harms of changing your medication?

**Probes**

- Were you given any options or alternative approaches to manage the change?

1. Please walk me through the process of going off of the medication, including what you thought went well or any challenges you had.

**Probes**

- Was the process difficult? If so, what made it difficult?
- What went well about the process (e.g, understanding that you could restart the medication, getting help from clinical staff or family members)?

9. Did the doctor/pharmacist support and guide you through the process? If so, how?

**Probes**

- Did your doctor/pharmacist follow-up with you and monitor your progress? If so, how?

10. Please tell us about anything could have done differently to make the process easier for you.

**Probes**:

- Has tapering off the medication changed how you feel or changed your health in some way that you can tell?

11. Is there anything about the process of stopping or going off of the medication that we didn’t ask you about that you would like to share or feel is important for us to know?

12. Are there people in your life that we should talk to who helped you manage the changes to your medications?

Great, thank you for sharing your experience I really appreciate your time and willingness to do this interview. I am going to turn off the audio recorder now and ask you to confirm your mailing address so that we can get the gift card sent out to you.

TURN OFF AUDIO RECORDER
